# Gas-Containing Disc Herniation at L1/2 Successfully Treated by Full-Endoscopic Transforaminal Discectomy: A Case Report

**DOI:** 10.7759/cureus.90995

**Published:** 2025-08-25

**Authors:** Yasushi Inomata, Ryoji Tominaga, Ryuichi Watanabe, Kento Takebayashi, Kazuyoshi Yanagisawa, Katsuhiko Ishibashi, Hisashi Koga, Hiroki Iwai

**Affiliations:** 1 Department of Orthopaedic Surgery, Iwai Orthopaedic Hospital, Tokyo, JPN; 2 Department of Neurosurgery, Iwai FESS Clinic, Tokyo, JPN; 3 Department of Neurosurgery, Iwai Orthopaedic Medical Hospital, Tokyo, JPN

**Keywords:** full-endoscopic discectomy, full-endoscopic lumbar discectomy, gas-containing lumbar disc herniation, transforaminal approach, upper lumbar disc herniation

## Abstract

Gas-containing lumbar disc herniation is a rare condition, with limited reports detailing its management. This case report presents the successful treatment of a gas-containing lumbar disc herniation using full-endoscopic discectomy via the transforaminal approach (FED-TFA) at an upper lumbar level, a challenging clinical scenario for which the application of this technique has been scarcely reported. An 81-year-old male patient with a complex surgical history involving multiple lumbar decompressions and spinal instrumentation presented with persistent numbness in the left lateral calf and progressively worsening pain in the left thigh. Magnetic Resonance Imaging and Computed Tomography scans confirmed a gas-containing disc herniation at L1/2. Conservative treatment was unsuccessful, and symptoms worsened significantly, prompting surgical intervention via FED-TFA. The patient's leg pain resolved immediately postoperatively; the visual analog scale score for leg pain improved from 6/10 preoperatively to 0/10 on postoperative day 1. This symptom relief was sustained, with no recurrence of leg symptoms at the one-year follow-up. This case highlights the potential of FED-TFA as an effective surgical option for treating refractory gas-containing lumbar disc herniation. Its key advantages include the ability to circumvent scarred posterior tissue while preserving posterior elements (such as the facet joint) and avoiding paraspinal muscle dissection, offering a critical "fusion-sparing" benefit. Furthermore, direct endoscopic visualization enables the thorough and controlled evacuation of intradiscal gas. These combined advantages make FED-TFA particularly suitable for complex cases at challenging spinal levels.

## Introduction

Gas-containing disc herniations (GCDHs) are rare spinal pathologies, typically observed in the lower lumbar region and associated with degenerative disc disease and the vacuum phenomenon [1,2]. Although spontaneous resolution of GCDH has been documented [3], persistent or progressive cases causing neurological symptoms often require intervention. Minimally invasive options such as Computed Tomography (CT)-guided aspiration have been described [4], but their efficacy can be limited by anatomical constraints or scar tissue from prior surgeries. Open surgery, while effective, is more invasive and may not be ideal for all patients [5].

This clinical challenge prompts a search for alternative approaches. For common lumbar disc herniations, full-endoscopic discectomy (FED) is an established method proven to be effective [6,7]. The full-endoscopic technique itself offers a pathology-specific advantage for GCDH, as direct visualization allows for effective evacuation of compressive epidural gas, thereby achieving neural decompression. Furthermore, the transforaminal approach (TFA) chosen in this case provides a critical "fusion-sparing" benefit by preserving posterior elements and paraspinal muscles while circumventing scar tissue from prior operations. Despite these combined advantages, the application of FED-TFA for treating GCDH in the upper lumbar spine remains scarcely reported [8,9].

We present a rare case of progressive L1/2 gas-containing disc herniation in an elderly patient with a complex surgical history, successfully treated with FED-TFA. This report highlights the distinct advantages of this technique in managing symptomatic GCDH, particularly in challenging clinical scenarios involving upper lumbar levels and a history of prior surgery.

## Case presentation

Patient information

An 81-year-old man presented with chronic left lower limb and lower back pain. His complex surgical history included multiple procedures at the L3/4 level, beginning with microendoscopic discectomies and culminating in posterior lumbar interbody fusions (PLIF) with subsequent revisions for complications, including infection. Notably, he had also undergone a microendoscopic laminotomy (MEL) at the L1/2 level three years earlier. His most recent surgery, one year before presentation, involved an MEL at L4/5 with concurrent removal of hardware from the prior L3/4 fusion. His medical history included hypertension and dyslipidemia.

Clinical presentation and preoperative course

One month after his most recent surgery (L4/5 MEL with hardware removal from the L3/4 level), the patient began experiencing numbness in his left lower limb. Physical examination at that time revealed a positive left Kemp’s test and diminished patellar and Achilles tendon reflexes. Straight leg raise and femoral nerve stretch tests were negative. Over the following year, he underwent a course of conservative treatment, which included continuous oral medications (non-steroidal anti-inflammatory drugs, pregabalin, and duloxetine) and a series of five left L2 nerve root blocks.

**Figure 1:**
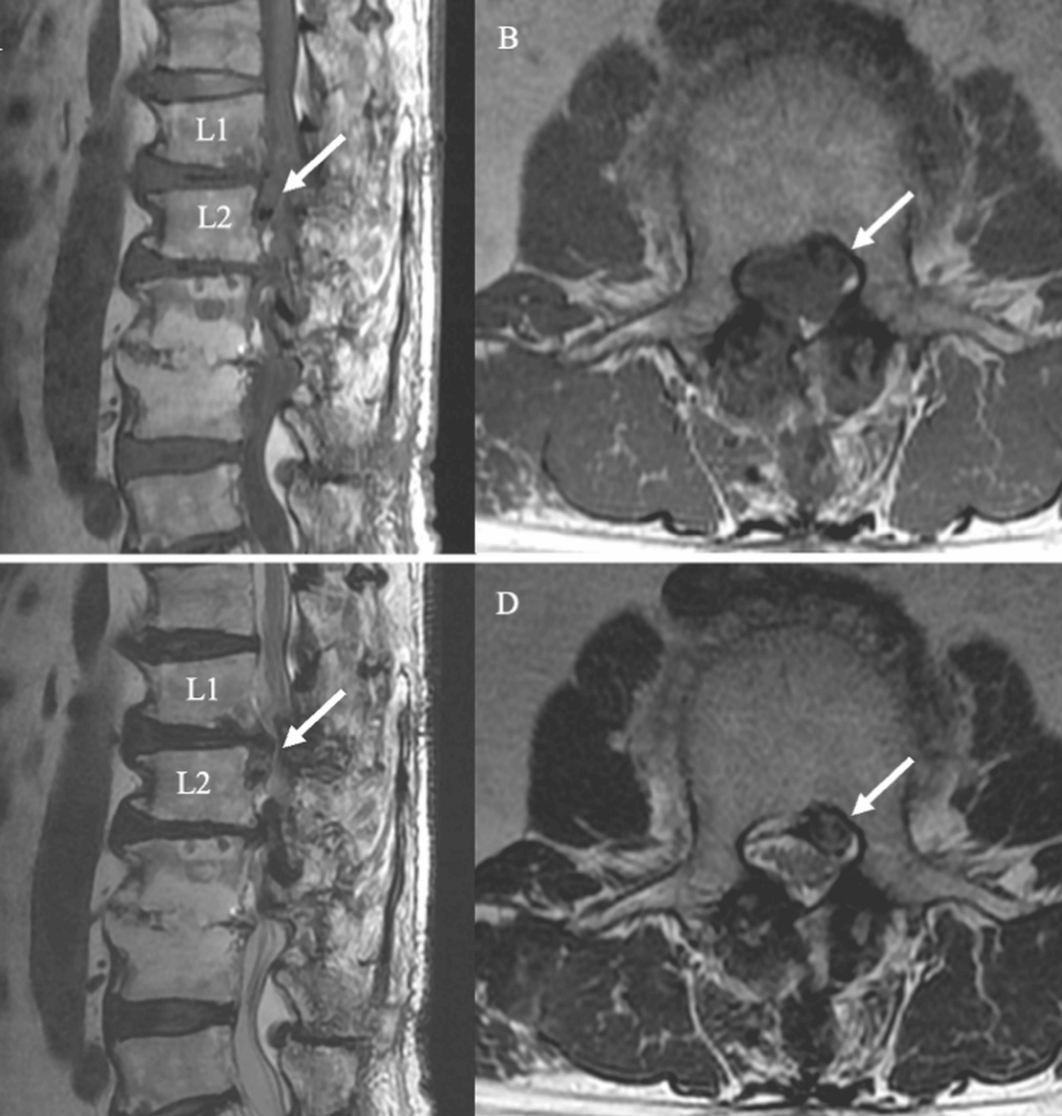
Initial MRI Findings of L1/2 Disc Herniation Sagittal and axial T1-weighted (A, B) and T2-weighted (C, D) MRI scans obtained 10 months before surgery show a small gas-containing disc herniation at the L1/2 level *(white arrow)*. The lesion appears as low signal intensity and mildly compresses the dural sac.

**Figure 2:**
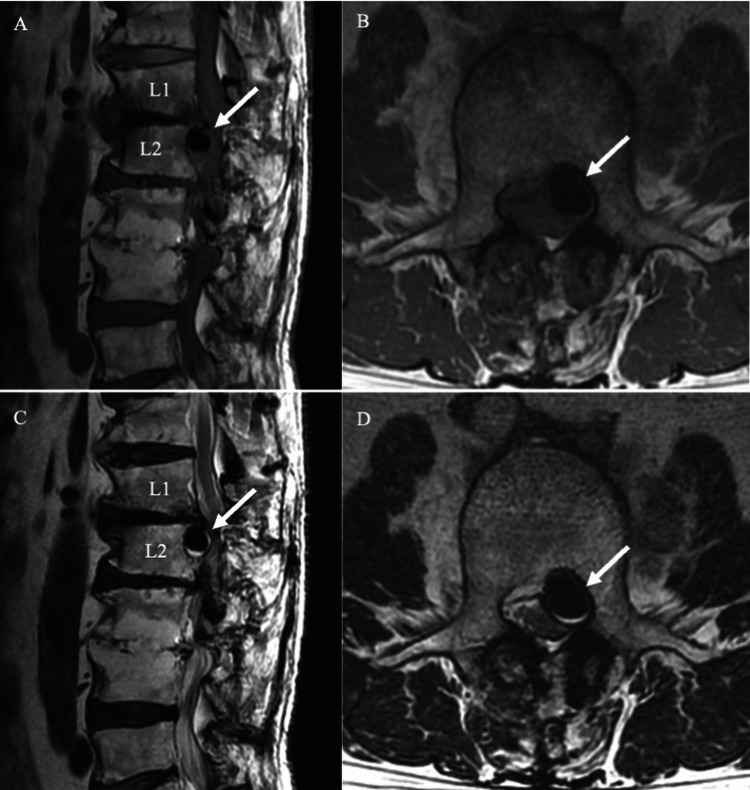
Progressive Enlargement of Gas-Containing Herniation Sagittal and axial T1-weighted (A, B) and T2-weighted (C, D) MRI scans obtained 18 days before surgery show significant enlargement of the epidural gas-containing lesion at L1/2 *(white arrow)*. The lesion compresses the thecal sac and the left L2 nerve root.

**Figure 3:**
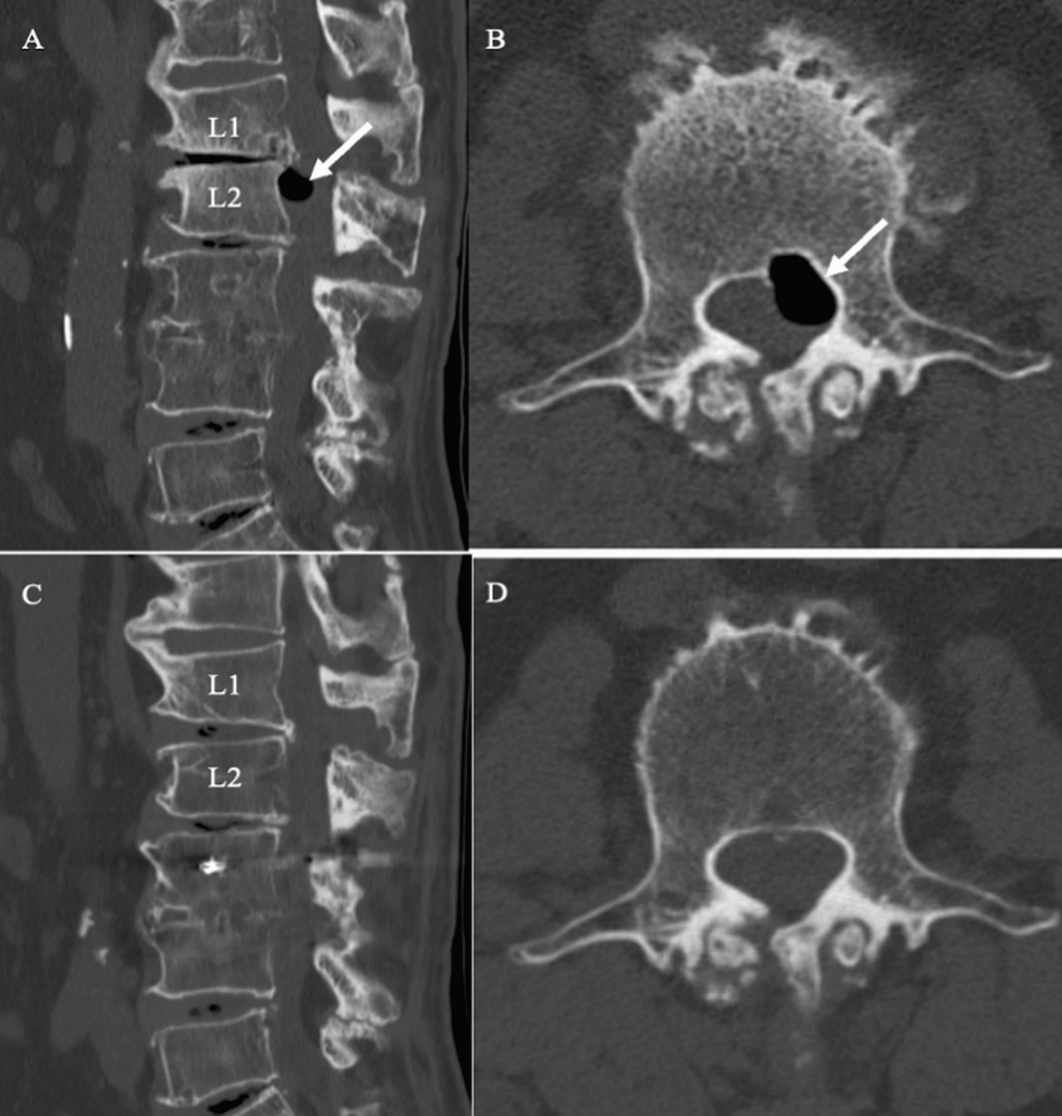
Preoperative CT Demonstrating Vertebral Scalloping and Epidural Gas Accumulation Sagittal (A) and axial (B) CT images obtained 18 days before surgery show an epidural gas-containing cyst causing posterior scalloping of the L2 vertebral body *(white arrow)*. For comparison, sagittal (C) and axial (D) CT images obtained 18 months earlier show no significant scalloping at that time.

Despite these measures, his symptoms progressively worsened, with severe left thigh and groin pain significantly impairing his daily activities. Magnetic resonance imaging (MRI) performed at the beginning of this one-year period revealed a small gas-containing disc herniation at L1/2 (Figure 1). A follow-up MRI a year later showed that the herniation had markedly enlarged, resulting in significant nerve root compression (Figure 2). Subsequent CT imaging confirmed a cystic lesion with gas accumulation and posterior scalloping of the vertebral body (Figure 3), which led to the decision for surgical intervention.

Therapeutic intervention

The patient was informed of all viable treatment options. These included continued conservative management, which had proven ineffective, and a more invasive revision posterior surgery. A decompression-only procedure via a conventional posterior approach was considered challenging, not only due to the significant scar tissue from the patient’s prior surgery at the same L1/2 level, but also because of the higher risk of iatrogenic facet joint violation inherent to the upper lumbar anatomy. A more extensive fusion surgery could also have been considered; however, the associated risks were deemed substantial given the patient’s advanced age, medical comorbidities, and history of postoperative infection. The third option was the proposed FED-TFA. After a thorough discussion of the potential risks and benefits of each approach, the patient opted for the minimally invasive endoscopic procedure.

Accordingly, FED-TFA was performed under general anesthesia with the patient in the prone position. An approximately 1-cm skin incision was made 7.5 cm lateral to the midline at the L1/2 level. A full-endoscope with a 7.9-mm outer diameter and a 4.2-mm working channel was used under gravity-fed saline irrigation.

To expose the cystic lesion while maximally preserving the facet joint, the superior articular process (SAP) and the cranial portion of the L2 pedicle were drilled. The precise trajectory and extent of this technically demanding bone removal were continuously monitored under biplanar C-arm fluoroscopy. The anteroposterior view was used to confirm the medial-lateral position of the drill relative to the pedicle, and the lateral view guided the drilling depth, ensuring adequate access without compromising pedicle integrity.

The exposed cyst was then bluntly punctured with a dissector to allow for initial passive gas egress. Subsequently, the working channel was used for active flushing with saline, which effectively evacuated the residual gas. Given the significant scarring from the prior surgery at this level, both intentional resection of the cyst wall and direct visualization of the nerve root were avoided to minimize the risk of iatrogenic injury. Successful decompression was confirmed indirectly by observing the collapse of the cystic lesion and the cessation of further gas outflow during irrigation. This entire process was recorded (Video 1). Written informed consent, including permission for publication of video material, was obtained from the patient preoperatively.

**Video 1:** Gas-Containing Lumbar Disc Herniation, L1/2 Full-Endoscopic Discectomy: Transforaminal Approach

Follow-up and outcomes

The patient experienced immediate and complete relief of his leg pain postoperatively; his Visual Analog Scale (VAS) score for leg pain improved from 6/10 preoperatively to 0/10 on postoperative day 1. This clinical improvement was maintained, with the patient remaining symptom-free at the one-year follow-up. A more comprehensive functional assessment using a questionnaire such as the Oswestry Disability Index could not be performed due to the 81-year-old patient’s inability to complete it, resulting from a combination of hand tremors and difficulty reading its fine print.

**Figure 4:**
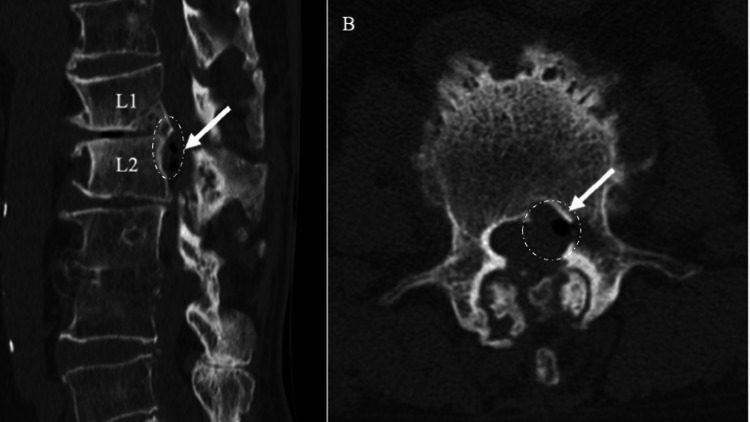
Immediate Postoperative CT Scan After FED-TFA Sagittal (A) and axial (B) CT images obtained on postoperative day 1 demonstrate a marked reduction of the epidural gas component *(*arrow and *dotted circle)*. FED-TFA: full-endoscopic discectomy via the transforaminal approach.

**Figure 5:**
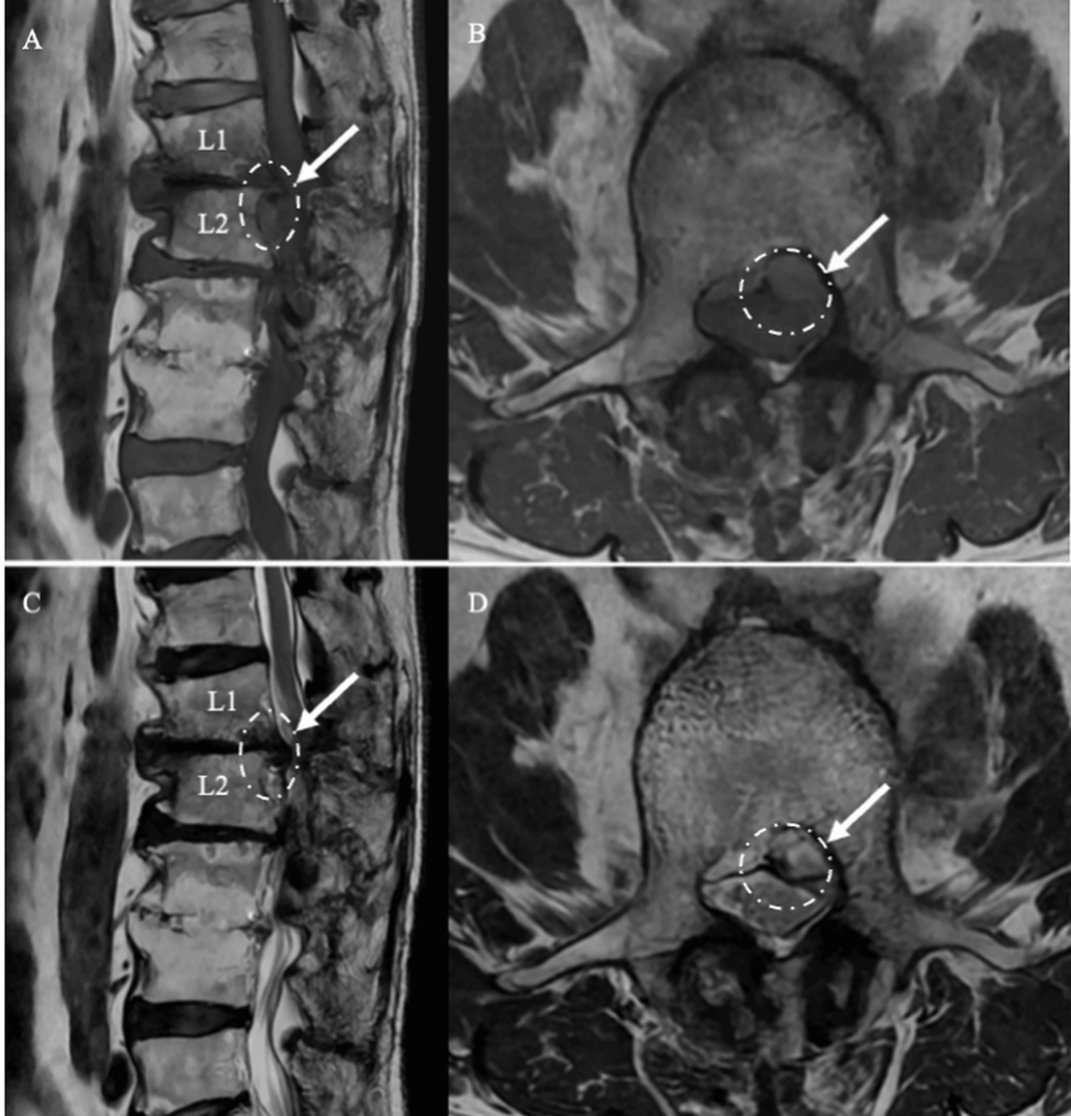
Postoperative MRI at Three Months Sagittal and axial T1-weighted (A, B) and T2-weighted (C, D) MRI scans obtained three months after surgery show a significant reduction in the gas component and sustained decompression of the nerve root at L1/2 *(white arrow *and dotted circle*)*.

**Figure 6:**
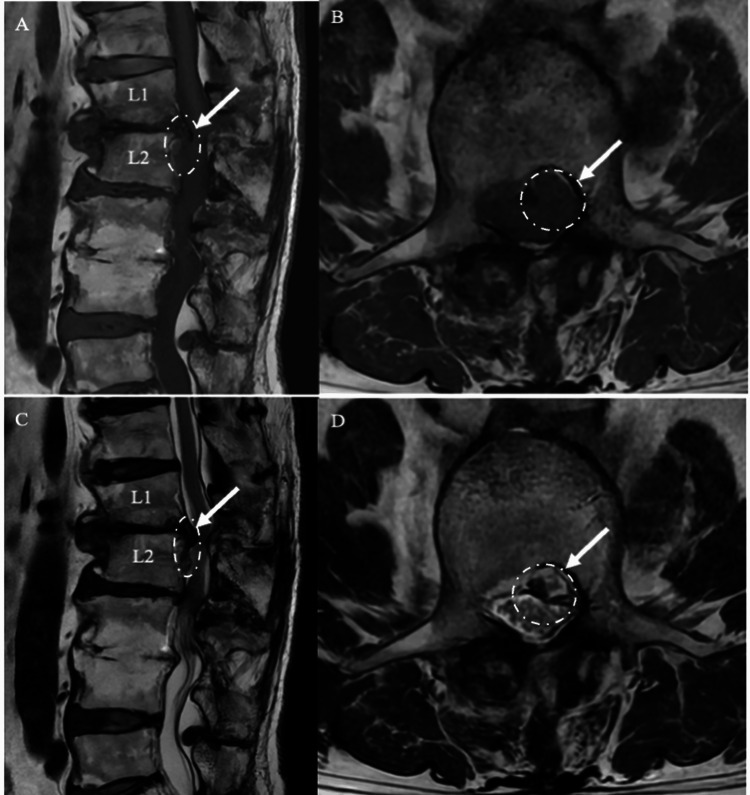
Postoperative MRI at One-Year Follow-up Sagittal and axial T1-weighted (A, B) and T2-weighted (C, D) MRI scans obtained one year after surgery demonstrate complete resolution of the epidural gas at the surgical site with no signs of recurrence *(white arrow *and *dotted circle)*.

Postoperative imaging corroborated this clinical outcome. A CT scan on postoperative day 1 demonstrated a marked reduction in the epidural gas component (Figure 4). Follow-up imaging at three months showed a significant reduction in gas and sustained decompression of the nerve root (Figure 5). At the one-year follow-up, MRI confirmed complete gas resolution at the surgical site (Figure 6).

## Discussion

GCDH is a rare condition characterized by the leakage of gas from a degenerated intervertebral disc through annular tears, resulting in the formation of a cyst-like lesion. A check-valve effect may contribute to persistent gas accumulation by allowing entry without adequate reabsorption [10,11]. In the present case, a small lesion gradually enlarged over several months and ultimately caused scalloping of the posterior vertebral body, suggesting persistent epidural gas accumulation.

GCDH typically occurs in elderly individuals with advanced disc degeneration, with most cases reported in the lower lumbar spine (e.g., L4/5 and L5/S1). It has been reported that affected patients ranged in age from 51 to 84 years (mean: 64.4 years), and all cases exhibited the vacuum phenomenon [1]. While prior surgery can accelerate adjacent segment degeneration, a direct causal link to the specific formation of GCDH is not well established in the literature, making our case (with a lesion at a previously operated level) noteworthy. In the present case, advanced degenerative changes, including the vacuum phenomenon, were also observed. On MRI, gas components generally appear as low-intensity signals on both T1- and T2-weighted sequences [12]. Although early MRI in our case did not reveal significant signal voids, subsequent CT imaging clearly demonstrated the gas-containing lesion, confirming the diagnosis.

Management strategies for GCDH vary depending on clinical severity and available resources. Although some cases resolve spontaneously, intervention is often required. CT-guided aspiration offers a less invasive alternative, but its effectiveness can be limited by recurrence [4,13,14]. Conventional posterior open surgery, whether decompression alone or with fusion, is another option, but it can be particularly challenging in certain scenarios. These include cases with a history of prior surgery at the same index level, as in our case, or upper lumbar pathologies where the anatomy increases the risk of iatrogenic facet joint violation. It is precisely in these situations that the TFA becomes advantageous, as it bypasses these compromised posterior structures entirely.

Reports on endoscopic treatment of GCDH are extremely limited. While successful endoscopic treatments for lower lumbar GCDH have been reported by Inokuchi et al. (via a TFA) [8] and more recently by Ioroi et al. (via an interlaminar approach) [9], our case is distinct as it involves an upper lumbar lesion in a patient with a history of prior surgery at the same level. This specific clinical scenario presented several technical challenges. First, a conventional posterior approach was considered high risk, primarily because the more sagittally oriented facet joints typical of the upper lumbar spine increase the risk of iatrogenic violation during decompression. The TFA was therefore particularly advantageous, as it bypassed these posterior structures entirely. Second, access to the herniated lesion was further complicated by the narrow foramen and vertebral scalloping, which necessitated extended drilling of the SAP and cranial pedicle. However, once access was achieved, intraoperative continuous irrigation and a flushing technique allowed for efficient evacuation of residual gas under direct visualization, addressing the core pathology.

Although the cyst wall was not intentionally resected, the patient remained symptom-free at the one-year follow-up. This durable result contrasts with recurrences reported after CT-guided aspiration. A key procedural difference that may have contributed to this distinction is the thorough saline irrigation performed under direct visualization, a component absent in simple aspiration. It is plausible that this flushing not only evacuates gas but also alters the local pressure dynamics, potentially promoting the sealing of any communication with the parent disc. However, it is impossible to determine from this single report whether this procedural element or the natural course of this specific case was responsible for the favorable outcome.

## Conclusions

FED-TFA appears to be a feasible and effective minimally invasive option for symptomatic GCDH. In complex cases, its key advantages are its ability to circumvent scarred posterior tissue while preserving posterior elements and paraspinal muscles, offering a "fusion-sparing" benefit. Furthermore, the technique allows for the thorough evacuation of intradiscal gas under direct visualization. This approach may therefore be particularly useful for challenging presentations, such as those involving the upper lumbar spine or a history of prior surgery at the index level. Further investigation through larger case series is warranted to establish the long-term efficacy and definitive role of FED-TFA in the treatment of GCDH.
